# Clinical outcomes of modified simple limbal epithelial transplantation for limbal stem cell deficiency in Chinese population: a retrospective case series

**DOI:** 10.1186/s13287-021-02345-2

**Published:** 2021-05-01

**Authors:** Yinghui Wang, Xiaodan Hu, Ke Yang, Yang Zhang, Shijing Deng, Zhiqun Wang, Shang Li, Lei Tian, Ying Jie

**Affiliations:** Beijing Institute of Ophthalmology, Beijing Tongren Eye Center, Beijing Tongren Hospital, Beijing Ophthalmology & Visual Sciences Key Laboratory, Capital Medical University, Beijing, 100730 China

**Keywords:** Simple limbal epithelial transplantation, Limbal stem cell deficiency, Limbal stem cells, In vivo confocal microscopy, Impression cytology

## Abstract

**Objective:**

To report the clinical outcomes of a novel surgical technique, namely simple limbal epithelial transplantation (SLET), for the treatment of limbal stem cell deficiency (LSCD).

**Methods:**

Thirteen patients (13 eyes) with LSCD who underwent autologous (10 eyes) or allogeneic (3 eyes) modified SLET between 2018 and 2021 were enrolled in this study. Grades of symblepharon, corneal conjunctivalization, vascularization, opacification, and visual acuity (VA) were evaluated preoperatively and postoperatively. In 2 cases, in vivo confocal microscopy (IVCM) and impression cytology (IC) were performed to assess the proliferation and degeneration of limbal tissue.

**Results:**

At a postoperative follow-up of 6.5±5.3 (range, 2–20) months, 10 (10/13, 76.92%) eyes maintained a successful outcome. The grades of symblepharon, corneal conjunctivalization, vascularization, and opacification were significantly improved after SLET (*P*<0.05). Two-line improvement in VA was found in 6 (6/10, 60%) eyes of the successful cases. Recurrence of LSCD occurred in 3 (3/13, 23.08%) eyes, and conjunctival cyst occurred in 1 patient. After SLET, the morphology and structure of corneal epithelial cells and epithelial transition around the limbal tissue fragments were detected by IVCM and IC.

**Conclusions:**

Our findings suggest that the SLET is a safe and effective technique for the treatment of LSCD. The corneal stroma and hAM can provide protection and nutrition for the limbal stem cells (LSCs) without negatively influencing the clinical outcomes. IVCM and IC after SLET can evaluate the effectiveness of surgery and the transition of LSCs and corneal epithelial cells.

## Background

Limbal stem cells (LSCs) reside in the basal epithelium of the limbus, which is the boundary between the cornea and conjunctiva [1]. The importance of LSCs in maintaining the homeostasis of corneal epithelium and wound healing has been confirmed in a variety of researches [2, 3]. Damage to the limbus, as a result of injury or disease, can lead to limbal stem cell deficiency (LSCD).

Clinical features of LSCD include corneal conjunctivalization, neovascularization, recurrent or persistent epithelial defects (PED), and scarring, leading to pain, impairment of vision, and may even progress to blindness [4]. LSCD can be categorized into 3 stages based on the extent of corneal and limbal involvement detected by clinical examinations [5]. There are limitations of slit-lamp examination in the diagnosis of LSCD owning to the signs of abnormal epithelium. Impression cytology (IC) and in vivo confocal microscopy (IVCM) may reflect more accurately the phenotype of the epithelium; thus, both are considered more sensitive in diagnosis [5, 6].

Treatment of LSCD has been challenging, especially in bilateral total LSCD, while limbal stem cell transplantation (LSCT) can reverse this condition [7]. The LSCT technique has evolved from conjunctival-limbal autografting (CLAu) to cultivated limbal epithelial transplantation (CLET) over the recent decades [8, 9]. In 2012, Sangwan et al. presented a novel surgical technique of limbal transplantation, namely simple limbal epithelial transplantation (SLET), which could combine the advantages of CLAu and CLET while eliminating the limitations of both earlier techniques [10]. The original technique describes a human amniotic membrane (hAM) graft that was placed over the bared ocular surface, 8–10 pieces of transplants; expanded in vivo over the hAM; and distributed around the center of the ocular surface. In 2014, sandwich therapy was proposed by AMESCUA, in which 2 hAM layers are used to wrap the LSCs [11]. According to different donor sources, SLET can be divided into autologous and allogeneic classes. Long-term results indicated that SLET is an effective and safe technique for treating LSCD, while the therapeutic efficacy of SLET is comparable with CLAu and CLET [12, 13].

Although there have been numerous case reports of SLET, no cases have been reported from China. No one has studied the migration and differentiation of limbal tissue on imaging. In the present study, hAM was used to cover and protect the harvested LSCs. To analyze the epithelial phenotypes of the cornea after SLET, IVCM and IC were performed in 2 patients postoperatively.

## Methods

### Study design and patients

This was a single-center, retrospective, interventional study. Thirteen eyes of 13 patients who were clinically diagnosed with LSCD underwent autologous (10 eyes) or allogeneic (3 eyes) modified SLET at the Capital Medical University of Beijing Tongren Hospital, Beijing, China, from April 2018 to February 2021. Of these, 1 patient received penetrating keratoplasty (PK) combined with SLET, 1 patient underwent oral mucosa transplantation combined with SLET, and 1 patient received PK at 16 months after SLET.

### Data collection

Data were collected on every visit, and the completed form was filed in the medical record. The patients’ demographic and clinical data included age, gender, etiology and date of injury, details of prior surgery, visual acuity (VA), intraocular pressure, details of intraoperative surgery, postoperative complications, duration of follow-up, and status of the ocular surface at each visit (Tables 2 and 3).

### Surgical technique

**Fig. 1:**
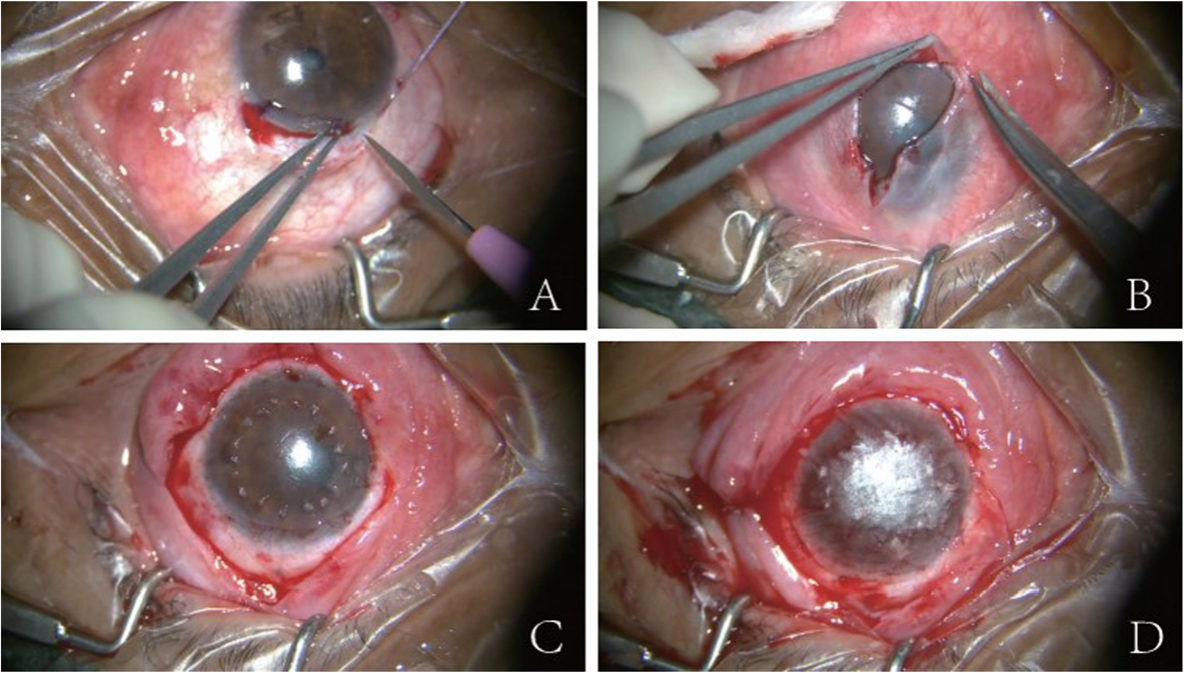
Intraoperative photographs showing the technique of modified SLET. **a** A 1 × 6 mm area of limbus was dissected from the donor’s eye. **b** The peritomy was performed, and the fibrovascular pannus was excised from the recipient ocular surface to expose the corneal stroma. **c** Ten to 15 pieces of grafts were placed on the ocular surface, sparing the visual axis. **d** The hAM was covered on the limbal tissue with 10-0 nylon sutures

In contrast to previous surgical procedures, we herein changed the order of limbal tissue and hAM and cut the limbal tissue into more tinier pieces. In brief, a 1 mm × 6 mm limbal biopsy was excised from the superior limbus of the donor’s eye and cut into 10–15 tiny fragments; peritomy was carried out; excise the fibrovascular pannus to expose the corneal stroma; the biopsied tissue placed on the corneal stroma, sparing the visual axis; and the hAM was then covered on the limbal tissue with fibrin glue or 10-0 nylon sutures (Fig. 1).

### Postoperative management and follow-up

Recipient eyes were prescribed tobramycin and dexamethasone eye drops (Alcon-Couvreur, Bornem, Belgium) and 0.1% sodium hyaluronate eye drops (URSAPHARM Arzneimittel GmbH, Saarbrücken, Germany) 4 times/day for 2 weeks. The bandage contact lens was removed from the recipient’s eye on day 14 after SLET. Prednisolone acetate ophthalmic suspension eye drops (Allergan Pharmaceuticals Ireland, Dublin, Ireland) were prescribed in tapering doses 4 times/day for 8–12 weeks. The donor’s eye was prescribed 0.1% sodium hyaluronate eye drops 4 times/day for 4 weeks. At every visit, VA was measured, and slit-lamp examination was undertaken. To confirm the epithelial phenotype postoperatively, IVCM and IC were performed in 2 cases.

### In vivo confocal microscopy

IVCM was performed using Heidelberg Retina Tomograph (HRT3) (Heidelberg Engineering GmbH, Heidelberg, Germany) postoperatively in case 3 and case 6. Before the examination, the recipient eye needed to be topically anesthetized by proparacaine hydrochloride eye drops (Alcon-Couvreur, Bornem, Belgium). A drop of Carbomer gel (Dr. Gerhard Mann Chem-Pharm. Fabrik GmbH, Hamburg, Germany) was applied to the disposable cap (TomoCap, Heidelberg Engineering GmbH, Heidelberg, Germany) and the cornea to protect the ocular surface. TomoCap was then installed on the corneal module and advanced gently to touch the cornea; shoot the center of the cornea, limbus, and limbal tissues; and take 3 or more images at each location.

### Impression cytology

IC was performed using multiple square Biopore membranes. The diameter of the membranes was 5 mm. Under topical anesthesia, the membranes were placed on the corneal surface for 2–5 s. The membranes were soaked in formalin to fix the color and then stained with periodic acid–Schiff (PAS). After that, photographs were taken with a microscope.

### Outcome measures

To objectively evaluate the condition of the ocular surface, the grade of symblepharon, grade of corneal vascularization, grade of conjunctivalization, and grade of corneal opacification were recorded preoperatively and postoperatively. The grading system was adapted from earlier publications (Table 1) [12, 14, 15]. For the success of SLET, the primary outcome was defined as a completely epithelialized, absence of neovascularization, and symblepharon invasion into the 5-mm diameter area of the central cornea. Meanwhile, the secondary outcome for success was the improvement in VA by 2 lines or greater. Failure was defined as the occurrence of PED, keratitis, progressive conjunctivalization, and vascularization.

**Table 1:** Scoring criteria of ocular surface appearance

Grade	Symblepharon	Corneal neovascularization	Conjunctivalization	Cornea opacification
0	No symblepharon	No neovascularization	No conjunctivalization	A clear cornea with clearly visible iris details
1	Limited to the conjunctiva	Confined to the limbus of the cornea	Conjunctivalization involving less than one quarter of the corneal surface	Partial obscuration of the iris details
2	Extending to the limbus	Extending up to the margin of the pupil	Conjunctivalization involving one quarter to one half	Poor visibility of the iris details with a barely visible pupil margin
3	Extending to the cornea	Extending beyond the margin of the pupil into the central cornea	Conjunctivalization involving more than one half of the corneal surface	Completely obscured iris and pupil details

### Statistical analysis

Statistical analysis was performed via the SPSS 26.0 software (IBM, Armonk, NY, USA). The Wilcoxon paired test was used for comparing the grade of the ocular surface between pre-operation and post-operation. *P* < 0.05 was considered statistically significant.

## Results

### Clinical outcomes

Thirteen eyes of 13 patients were included in the study (1 female, 12 males; mean age, 36.4±15.9 years old; range, 11–64 years old). Alkali injury (9/13, 69.23%) was found as the most common cause of LSCD. Patient demographics characteristics at baseline are summarized in Table 2. Among the 13 patients, 3 patients with bilateral LSCD underwent allogeneic-SLET (allo-SLET), and the remaining patients with unilateral LSCD underwent autologous-SLET (auto-SLET) (Table 3). The mean duration of follow-up was 6.5±5.3 (range, 2–20) months. At the final follow-up after SLET, the primary successful outcomes were observed in 10 (10/13, 76.92%) eyes, which maintained a successfully regenerated stable corneal surface without PED, progressive conjunctivalization, or vascularization. Among the successful cases, 8 (8/10, 80%) eyes underwent auto-SLET, while 2 (2/3, 66.67%) eyes underwent allo-SLET. Postoperatively, a statistically significant improvement was found in the grades of symblepharon (*Z*=−2.565, *P*=0.010), corneal conjunctivalization (*Z*=−2.913, *P*=0.004), vascularization (*Z*=−2.623, *P*=0.009), and opacification (*Z*=−2.414, *P*=0.016) (Table 4). Besides, a significant improvement in VA was observed in 8 (8/13, 61.5%) eyes, of which, successful clinical outcomes were achieved in 6 (6/10, 60%) eyes.

**Table 2:** Demographic and clinical characteristics of the patients before SLET

Case	Age (years old)	Sex	Eye	Etiology of LSCD (times)	Previous of surgery	Pre-op VA	Ocular surface appearance				
							Symblepharon (grade)	Vascularization (grade)	Conjunctivalization (grade)	Corneal opacification (grade)	Others
1	49	M	OD	Thermal injury (1.5 years)	AMT	HM	3	3	3	3	None
2	64	M	OD	Alkali burn (20 years)	None	HM	2	2	2	2	ED
3	37	M	OD	Alkali burn (7 months)	None	20/400	3	3	3	3	None
4	12	F	OD	Alkali burn (6 years)	None	20/63	1	2	3	1	None
5	33	M	OS	Alkali burn (5 months)	AMT	LP	3	2	3	3	Corneal central thinning
6	51	M	OS	Alkali burn (2 months)	AMT	20/1000	0	2	0	2	None
7	32	M	OD	Alkali burn (24 years)	None	LP	2	2	0	3	None
8	19	M	OS	Alkali burn (7 months)	AMT	LP	2	3	2	2	None
9	38	M	OS	Alkali burn (3 months)	Anterior chamber washout, entropion correction	20/100	0	1	2	1	None
10	36	M	OD	Thermal injury (1 years)	AMT	HM	3	3	3	3	Lower eyelid retraction, lower palpebral conjunctival defect
11	31	M	OS	Acid burn (6 months)	AMT	20/200	3	2	2	1	None
12	11	M	OS	Blast injury (2 years)	None	20/2000	0	3	3	3	None
13	60	M	OD	Alkali burn (8 months)	None	HM	3	3	3	3	None

*M*, male; *F*, female; *LSCD*, limbal stem cell deficiency; *Pre-op*, preoperative; *VA*, visual acuity; *OD*, right eye; *OS*, left eye; *AMT*, amniotic membrane transplantation; *LP*, light perception; *HM*, hand motion; *ED*, epithelial defect

**Table 3:** Outcomes and complications of patients who underwent SLET

Case	Surgery	Follow (months)	Post-op VA	Complications	Ocular surface appearance					Clinical result
					Symblepharon (grade)	Vascularization (grade)	Conjunctivalization (grade)	Corneal opacification (grade)	Others	
1	Auto-SLET	14	HM	None	1	2	1	3	Corneal degeneration, pre-existing deep vascularization	S
	PK+ECCE+IOL	3	20/400	None	0	0	0	0	None	S
2	Auto-SLET	6	20/63	None	0	1	1	1	None	S
3	Auto-SLET	20	20/50	Conjunctival cyst	0	2	1	1	None	S
4	Auto-SLET	3	20/40	None	1	2	0	1	Pre-existing deep vascularization	S
5	Allo-SLET	3	HM	None	2	1	0	3	None	S
6	Auto-SLET	5	20/63	None	0	1	1	1	Pre-existing deep vascularization	S
7	Auto-SLET	7	Fc/0.2m	Recurrent LSCD	2	1	1	3	None	F
8	Auto-SLET	3	20/40	None	0	1	1	0	None	S
9	Auto-SLET	2	20/40	Recurrent LSCD	0	1	1	1	Hyperemia of conjunctiva	F
10	Allo-ST+Auto-OMT+Auto-SLET	3	HM	Recurrent LSCD	1	3	3	3	None	F
11	Auto-SLET	2	20/400	None	0	1	0	0	None	S
12	Allo-SLET	8	20/1000	None	0	2	0	2	Pre-existing deep vascularization	S
13	PK+Auto-SLET	8	20/67	None	0	0	0	0	None	S

*Auto-SLET*, autologous simple limbal epithelial transplantation; *PK*, penetrating keratoplasty; *ECCE*, extracapsular cataract extraction; *IOL*, intraocular lens; *Allo-SLET*, allogeneic simple limbal epithelial transplantation; *Allo-ST*, allogeneic scleral transplantation; *Auto-OMT*, autologous oral mucosa transplantation; *Post-op*, postoperative; *VA*, visual acuity; *LSCD*, limbal stem cell deficiency; *HM*, hand motion; *Fc*, figure count; *F*, failure; *S*, success

**Table 4:** Grading of the ocular surface before and after surgery

	Symblepharon	Vascularization	Conjunctivalization	Corneal opacification
*Z*	−2.565	−2.913	−2.623	−2.414
*P*	0.010*	0.004*	0.009*	0.016*

*P<0.05

**Fig. 2:**
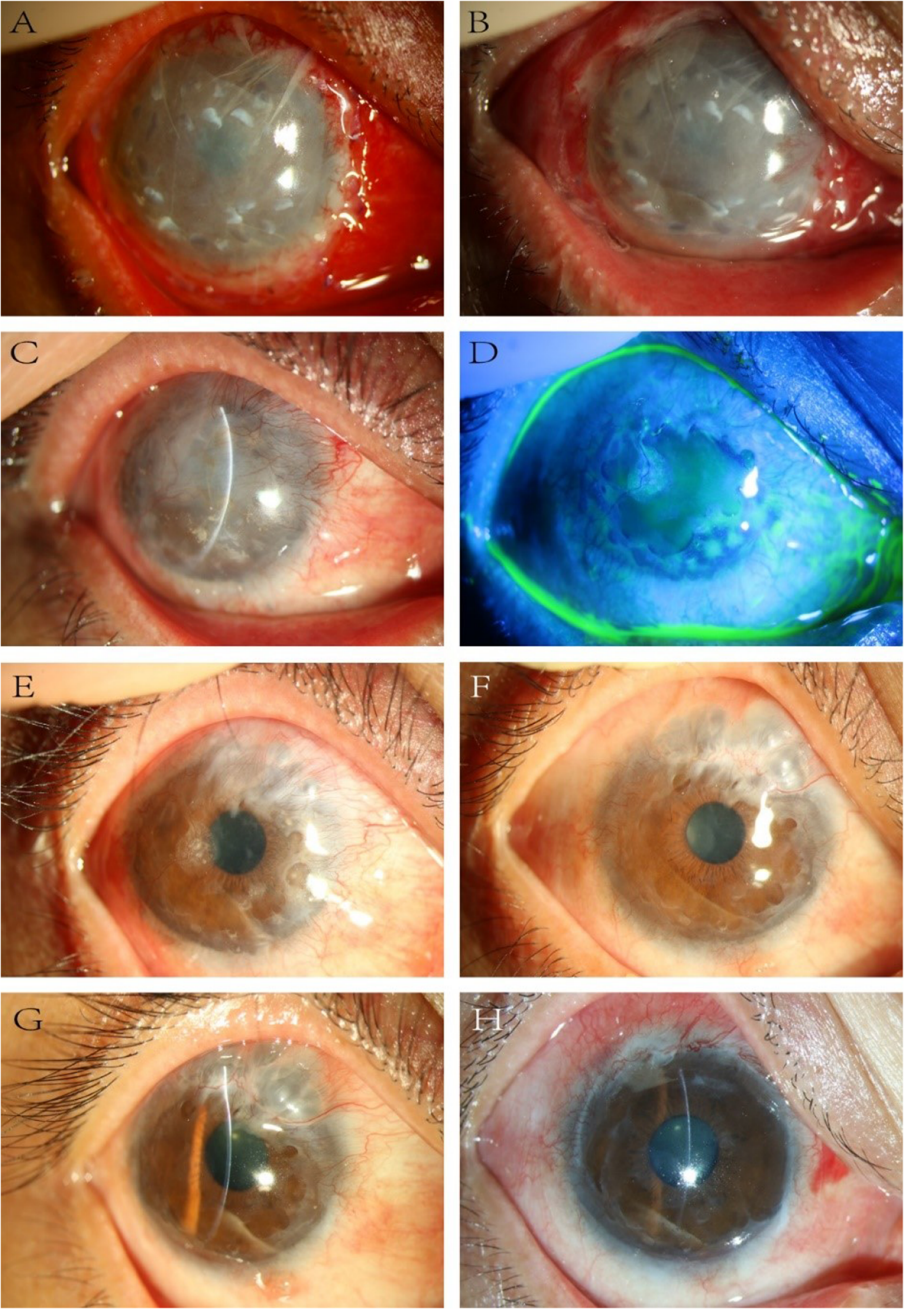
Longitudinal slit-lamp examination indicating the progressive disappearance of limbal biopsy fragments and the emergence of complications after SLET. **a** At 1 week after SLET, the hAM and the limbal biopsy fragments were evident. **b** After 1 month, the hAM was partially absorbed, and the limbal biopsy fragments appeared with more indistinct margins. **c** After 2 months, few reduced-sized fragments were visible. **d** After 3 months, fluorescein staining showed the smooth surface of the cornea, which indicated that the corneal was epithelialized. **e**–**g** After 4, 13, and 19 months, the grafts gradually disappeared, the cornea became clearer, and the conjunctival cyst appeared and grew. **h** After 20 months, the cornea was clear after cystectomy

There were no complications in the donor’s eyes. Recurrence of LSCD was the most common complication in 3 recipient eyes (3/13, 23.08%). In case 3, a conjunctival cyst was detected at 4 months postoperatively and gradually increased; thus, the patient underwent cystectomy at 19 months postoperatively. The long-term clinical outcomes of case 3 are shown in Fig. 2.

### IVCM and IC

**Fig. 3:**
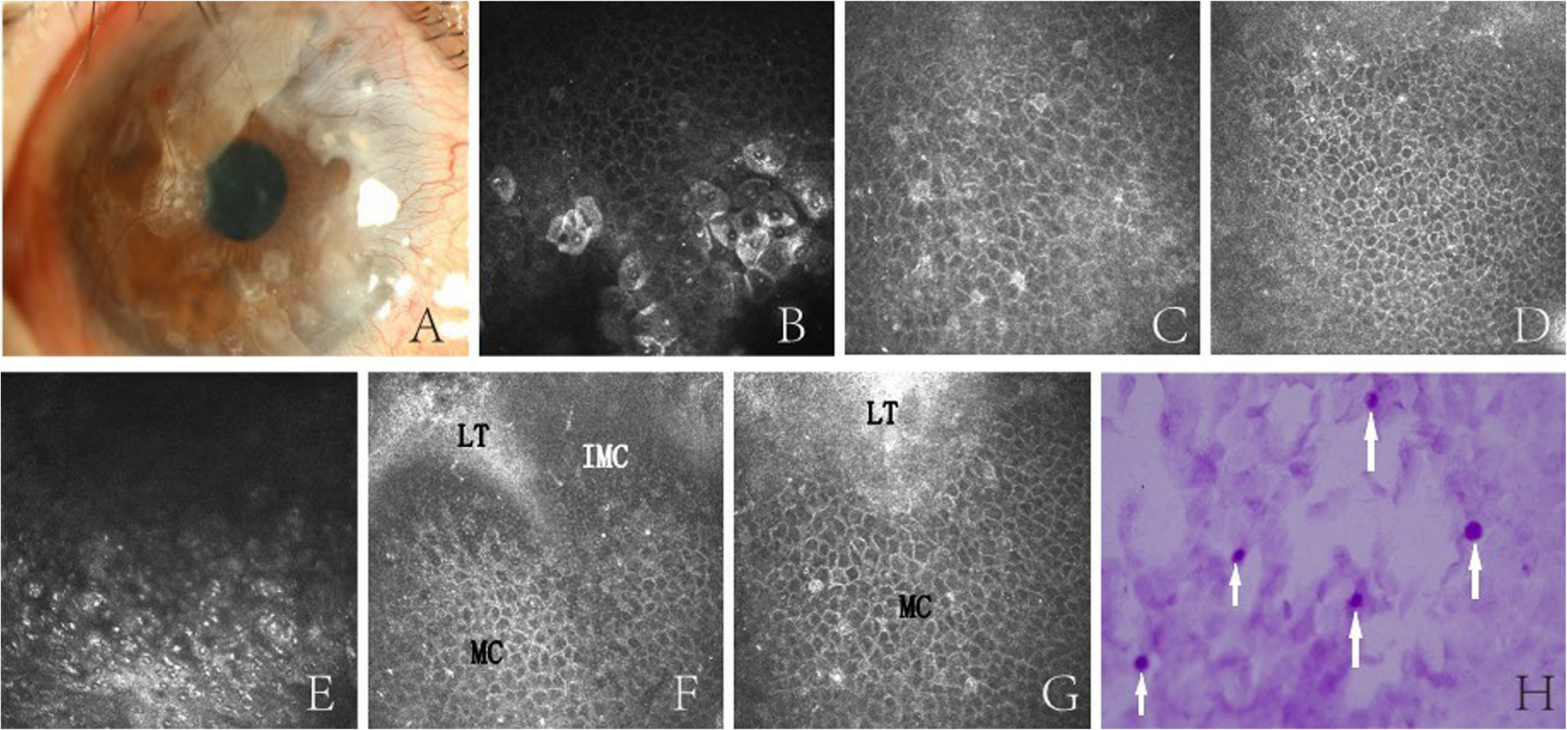
Representative images of case 3, in which epithelial regeneration was observed by IVCM and IC. **a** Slit-lamp biomicroscopy showing that the central cornea is transparent, with mild vascularization and conjunctivalization in the upper and nasal quadrants, and few implants are still visible. **b**–**d** Superficial polygonal cells, intermediate wing cells, and smaller basal cells in the central part of the cornea. **e** Palisade-like structures were not detected in the limbal tissue. **f** Immature epithelial cells with small indistinct cell borders, bright nuclei, and a high nucleus-to-cytoplasm ratio that gradually proliferated and migrated into mature cells. **g** Mature epithelial cells with hyper-reflective and defined borders were formed around the graft. **h** Goblet cells (arrows) with strong PAS staining were distributed in the upper quadrant of polygonal corneal epithelial cells in IC. The frame size is 400 × 400 μm. LT, limbal tissue; IMC, immature cells; MC, mature cells

IVCM and IC were performed on case 3 to investigate the epithelial phenotype 4 months after SLET. Vascularization and conjunctivalization in the upper and nasal quadrants could be detected by slit-lamp examination. IVCM showed the presence of a multilayered corneal-type epithelium in the central cornea, and the normal Vogt structure was not detected in the limbus. The proliferation and differentiation abilities of each limbal tissue were different. The intact epithelium was formed around some grafts, and the epithelial cells around some grafts were immature. The result of IC was consistent with that of the slit-lamp examination, which showed a small number of goblet cells were distributed in the upper quadrant of corneal epithelial cells (Fig. 3).

**Fig. 4:**
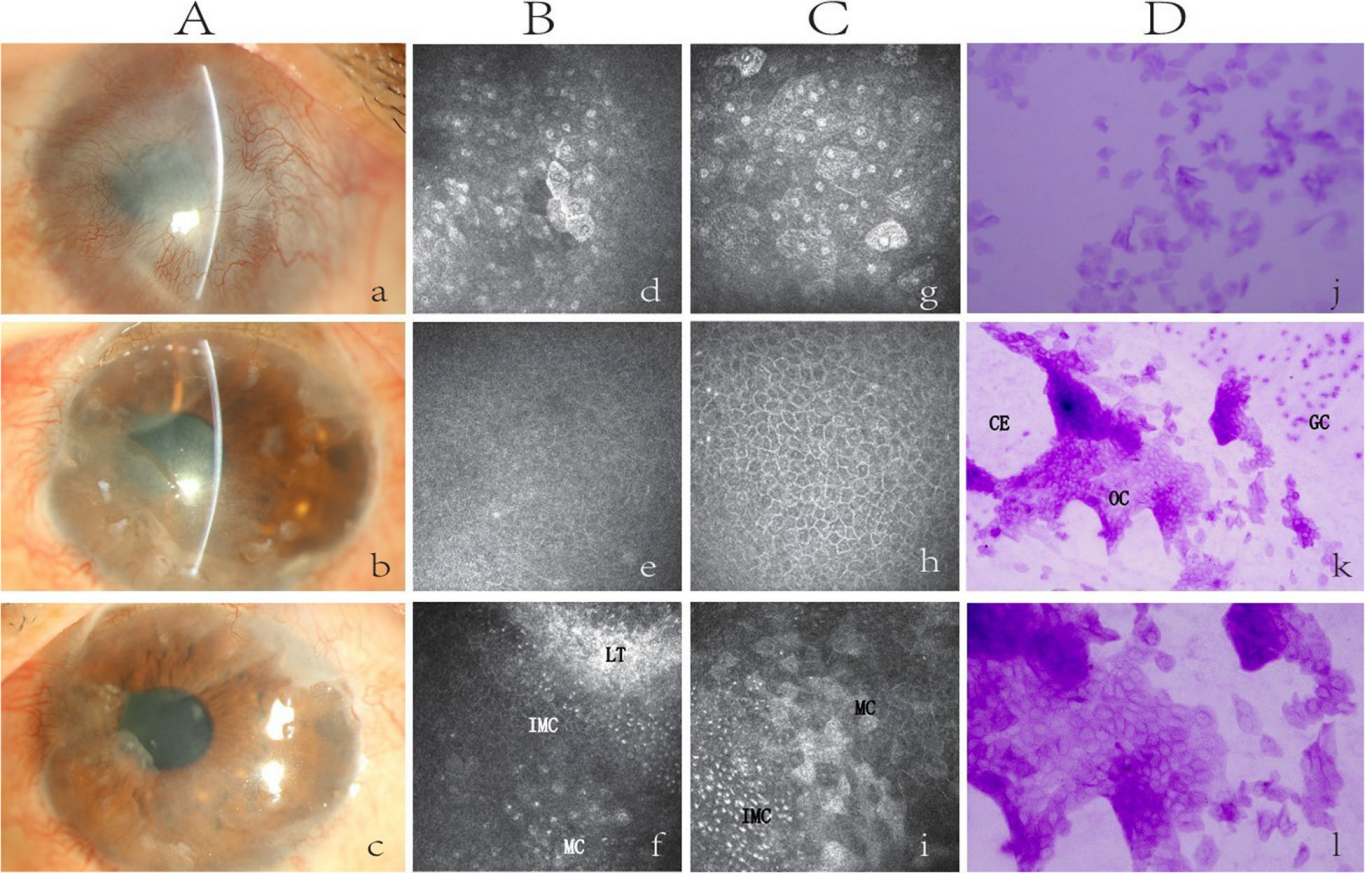
The epithelial analysis was performed at 3 (**B**) and 5 (**C**, **D**) months after SLET on case 6. **a** Preoperative image. **b** After 3 months, the hAM was almost absorbed, and the limbal tissues appeared with indistinct margins. **d**, **e** The epithelial cells of the central cornea appeared with indistinct cell borders. **f** The implants were mainly surrounded by immature epithelium. **c** After 5 months, the implants were almost invisible. **g**, **h** IVCM demonstrated that multilayered corneal epithelial cells were intact without goblet cells. **i** The area of immature cells around the implants was gradually reduced and replaced by a large number of mature cells. **j** IC showed a corneal phenotype without evidence of goblet cells. **k**, **l** The residual limbal tissue, containing a large number of ovoid cells from the donor’s eye, was surrounded by the corneal epithelial cells, which were separated from the conjunctival goblet cells. The frame size is 400 × 400 μm. LT, limbal tissue; IMC, immature cells; MC, mature cells; CE, cornea epithelium; OC, ovoid cells; GC, goblet cells

In case 6, IVCM was performed at 3 and 5 months after SLET. IC was carried out at 5 months postoperative. After SLET, the slit-lamp examination showed that the cornea was gradually transparent and the limbus tissues were gradually invisible. In IVCM, the boundary of the epithelial cells became clearer, and the volume of the cells gradually increased. The multiplication and differentiation of LSCs into immature epithelial cells could be observed, and the limbal tissues showed normal features of the superficial transition of the epithelia, which indicated that the immature epithelial cells around the tissues were gradually differentiated and matured. In IC, there was no goblet cell infiltration, while some limbal tissue fragments remained in the corneal epithelium (Fig. 4).

## Discussion

SLET is a relatively novel treatment for LSCD, which has long-term efficacy with a success rate of 76%, and 67% of successful cases attained 20/60 or a better vision [12]. In two other large-sized studies, the success rate of SLET was 70% and 83.8%, and 70% and 64.7% of patients gained two or more lines of improvement of VA [16, 17]. The success rate of allo-SLET is lower than that of auto-SLET, because it cannot avoid lysis of the implant due to immune rejection [18]. In the present study, primary successful outcomes were observed in 10 (76.92%) eyes. The success rates of allo-SLET and auto-SLET were 80% and 66.67%, respectively. Besides, 60% of successful cases gained a significant improvement in VA. Compared to previous studies, the surgical success rate of the modified SLET was similar, whereas the rate of improvement in VA was not remarkable. This is related to the fact that patients included in this study had severe corneal opacity, and SLET could restore the structure and function of the corneal epithelium, while it could not treat the residual corneal stromal opacity.

In traditional SLET, hAM supports the graft regeneration and acts as a scaffold for regenerating epithelium emerging out of the limbal tissue explants. In the current study, the limbus tissue directly contacted the corneal stromal, which indicated that the corneal stromal and hAM wrapped the LSCs and provided an in vivo medium for it. In addition, hAM has the effect of promoting tissue epithelialization, inhibiting the formation of new blood vessels and scars, and anti-inflammatory, which has been considered as one of the important biomaterials to promote corneal regeneration and to heal the wound [19, 20]. According to a previous study, from day 3 after amniotic membrane transplantation (AMT), hAM epithelium showed progressive signs of degradation, becoming undetectable at day 15, and hAM tissues were no longer detectable after 8 weeks [21]. In a case report presented by Chaudhuri et al., anterior segment optical coherence tomography (AS-OCT) was administered at the first 2 weeks for a patient who underwent SLET and showed that hAM settled down and adhered to the cornea on day 10 after SLET, while there was a complete epithelialization on the limbal side on day 14 [22]. We found that the time of hAM dissolution coincides with the time of corneal epithelialization. Therefore, we speculate that when the hAM is placed under the limbal tissue, the application and dissolution of hAM may affect the regeneration rate of the limbal tissue. The use of hAM as a soft contact lens can take the advantage of its biological features, while avoiding the influence of its dissolution on epithelial regeneration.

To our knowledge, the corneal stroma has a nutritional effect on LSCs; thus, the hAM is not used as a supporting substance. A variety of cellular products in the stroma, including growth factors/cytokines, extracellular matrix (ECM) components, and kinases, can support normal corneal development and homeostasis [23]. After wound healing, corneal epithelial cells release interleukin-1α (IL-1α) and interleukin-1β (IL-1β), accelerating the epithelial coverage of the wound [24]. Lumican in ECM maintains corneal transparency and promotes corneal epithelial wound healing by modulating the synthesis of collagen fibrils [25]. IKKβ (inhibitor of NF-κB [nuclear factor κB] kinase β) in keratocytes is required for corneal epithelial wound healing via repression of oxidative stress and attenuating hepatic fibrogenesis. Besides, corneal stromal stem cells have a therapeutic effect and can restore the ECM organization and corneal transparency in vivo [26]. Therefore, corneal stroma can serve as a potential nutritional source for the proliferation, motility, and differentiation of LSCs and cornea.

The renewal of the corneal epithelium and the differentiation of LSCs can be recognized by IVCM and IC based on the cell morphology and limbal tissue modifications. In agreement with previous researches, multi-layered corneal epithelia without conjunctival epithelia intruded in the central cornea were mainly detected in succeeded SLET [27]. A number of scholars suggested that during corneal healing, cells first repopulate the limbus and, then, heal centripetally [28]. However, in the current research, limbal crypts or palisade-like structures were not detectable in our patients. We found that after SLET, immature corneal epithelial cells and epithelial transition zone appeared around the limbus tissue, and the area of mature epithelium gradually increased over time. This finding is consistent with Mittal et al.’s result [29]. We can therefore speculate that each tissue may form an island of epithelial cells. In the early postoperative period, the islands are surrounded by immature epithelial cells, which can be produced by LSCs, and then, the cells gradually proliferate and differentiate into mature epithelium, and the area of ​limbal tissues gradually decreases.

The expansion of the epithelial cell islands makes epithelialization of the cornea; thus, we cut the transplants into more small pieces to obtain more cell islands. However, we found that different tissues grew at different rates, probably due to the different sizes of limbal explants. Kethiri et al. demonstrated that a minimal amount of 0.3-mm^2^ live tissue or ≥ 0.5-mm^2^ cadaver tissue would be sufficient for the expansion of LSCs in vitro [30]. Hence, surgery should make a balanced relationship between the number and the size of corneal tissue fragments, in order to ensure optimal tissue area, while increasing the number of epithelial cell islands. In contrast to other reports, to obtain more limbal tissues with a sufficient size, we expanded the sampling area of the limbus in the donor’s eyes as in CLAu, while no complications appeared in the donor’s eyes.

Our team first reported the application of SLET in China and achieved promising clinical results. In the present study, a conjunctival cyst was first reported in the recipient eye, which is a rare postoperative complication. It may be due to implantation of conjunctival epithelial cells into the subconjunctiva during surgery, followed by proliferation of epithelial cells and degeneration of the central part, or neoformative corneal epithelial cells may grow inward and extend into the subconjunctiva and degenerate in the central part, thereby forming a cyst. However, it could be removed by a simple resection with a good outcome.

The limitations of this study included small sample size, short follow-up period in some patients, and only 2 patients analyzed the epithelial morphology. Accordingly, the extension of sample size, a longer observation period, and performing IVCM and IC in pre- and post-operation are required to confirm our findings.

In conclusion, modified SLET seems to be a safe and effective technique for treating LSCD. The corneal stroma and hAM wrapped around the graft can provide protection and nutrition to the LSCs without negatively influencing clinical outcomes. IVCM and IC are highly significant to diagnose LSCD and characterize the healing process on the ocular surface after SLET.

## Data Availability

All data generated or analyzed during this study are included in this published article.

## Abbreviations

LSCs: Limbal stem cells
LSCD: Limbal stem cell deficiency
PED: Persistent epithelial defects
IC: Impression cytology
IVCM: In vivo confocal microscopy
LSCT: Limbal stem cell transplantation
CLAu: Conjunctival-limbal autografting
CLET: Cultivated limbal epithelial transplantation
SLET: Simple limbal epithelial transplantation
hAM: Human amniotic membrane
PK: Penetrating keratoplasty
VA: Visual acuity
PAS: Periodic acid–Schiff
auto-SLET: Autologous simple limbal epithelial transplantation
allo-SLET: Allogeneic simple limbal epithelial transplantation
AMT: Amniotic membrane transplantation
AS-OCT: Anterior segment optical coherence tomography
HGF: Hepatocyte growth factor
KGF: Keratinocyte growth factor
